# Detection of Anomalous Noise Events on Low-Capacity Acoustic Nodes for Dynamic Road Traffic Noise Mapping within an Hybrid WASN

**DOI:** 10.3390/s18041272

**Published:** 2018-04-20

**Authors:** Rosa Ma Alsina-Pagès, Francesc Alías, Joan Claudi Socoró, Ferran Orga

**Affiliations:** GTM—Grup de recerca en Tecnologies Mèdia, La Salle—Universitat Ramon Llull, Quatre Camins, 30, 08022 Barcelona, Spain; falias@salleurl.edu (F.A.); jclaudi@salleurl.edu (J.C.S.); forga@salleurl.edu (F.O.)

**Keywords:** low capacity, low cost, hybrid wireless acoustic sensor network, real-time signal processing, anomalous noise event, noise, road traffic noise, dynamic noise mapping, μController, μProcessor

## Abstract

One of the main aspects affecting the quality of life of people living in urban and suburban areas is the continuous exposure to high road traffic noise (RTN) levels. Nowadays, thanks to Wireless Acoustic Sensor Networks (WASN) noise in Smart Cities has started to be automatically mapped. To obtain a reliable picture of the RTN, those anomalous noise events (ANE) unrelated to road traffic (sirens, horns, people, etc.) should be removed from the noise map computation by means of an Anomalous Noise Event Detector (ANED). In Hybrid WASNs, with master-slave architecture, ANED should be implemented in both high-capacity (Hi-Cap) and low-capacity (Lo-Cap) sensors, following the same principle to obtain consistent results. This work presents an ANED version to run in real-time on μController-based Lo-Cap sensors of a hybrid WASN, discriminating RTN from ANE through their Mel-based spectral energy differences. The experiments, considering 9 h and 8 min of real-life acoustic data from both urban and suburban environments, show the feasibility of the proposal both in terms of computational load and in classification accuracy. Specifically, the ANED Lo-Cap requires around 16 of the computational load of the ANED Hi-Cap, while classification accuracies are slightly lower (around 10%). However, preliminary analyses show that these results could be improved in around 4% in the future by means of considering optimal frequency selection.

## 1. Introduction

Living with continuous exposure to high levels of traffic noise has been found to be harmful to human health, as it affects the quality of life of people living in urban and suburban areas [1]. Several actions have been conducted to address this problem, based on the European Noise Directive 2002/49/EC (END) [2] and the consequent strategic noise mapping assessment CNOSSOS-EU [3], which are the main requirements of current European legislation which request Member States to elaborate specific action plans to mitigate noise pollution, as well as making the public aware of the dangers of noise pollution.

Generally, until recently, noise measurements in cities have been conducted by professionals, who record and analyze the data in specific locations and time periods by using certified sound level meters. Subsequently, noise maps are generated from these noise level measurements by means of the application of complex acoustic models after data post-processing. These maps should be updated and published every five years to fulfill the END requirements for agglomerations with more than 100,000 inhabitants, major roads, major railways and airports [2]. However, this approach becomes difficult to scale when more measurements and/or locations are needed, besides the questionable representativity of the data and the subsequent lack of accuracy of this kind of predictive models. Internet of Things and Smart City frameworks have led to a change of paradigm for the city noise monitoring and management by means of Wireless Acoustic Sensor Networks (WASNs) [4]. In the last decade, several works focused on the design and implementation of WASNs for environmental noise monitoring have been proposed [5]. The main goal of these approaches has been to develop affordable solutions, maintaining the reliability of the acoustic measures, while improving the scalability of the system through optimum network design. Some WASN-based systems have been developed and tested across Europe, such as the IDEA project in Belgium [6] and the Cense project [7] in France, or the `Barcelona noise monitoring network’ in Spain [8], which follow quite similar approaches. Moreover, it is worth mentioning the SONYC project [9] in the USA, aimed at monitoring noise pollution in New York City, besides providing an accurate description of the surrounding acoustic environment.

The initial development of WASN in several cities has opened several challenges [10,11], especially those derived from acoustic signal processing. As a first step, the aforementioned approximations are only focused on global noise monitoring, without taking into account the type of traffic or the detection of specific acoustic events in the acoustic environment; issues which are mandatory to satisfy the END requirements [2]. In order to provide public bodies with reliable measurements of the noise caused by Road Traffic Noise (RTN) affecting citizens, events not related to RTN—denoted as Anomalous Noise Noise Events (ANE)—should be removed from the noise map computation. In this context, Acoustic Event Detection (AED) algorithms have been designed for several domains of application in urban environments, most of them developed within surveillance applications, which include noise source identification [12,13,14], together with first works focused on the separation between target and interfering signals for noise monitoring in cities [15,16].

**Figure 1:**
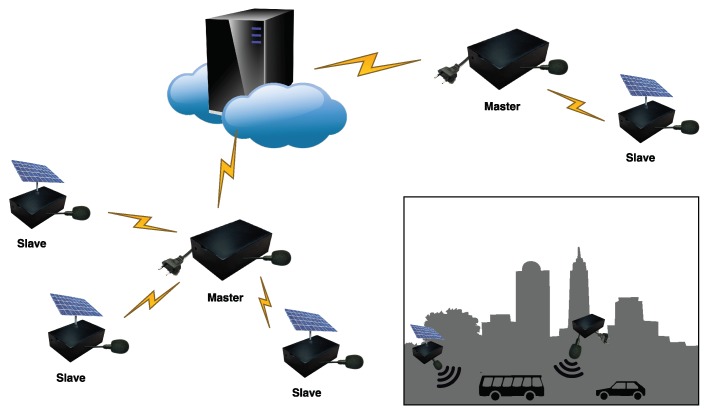
Scheme of an hybrid WASN architecture using Hi-Cap master sensor nodes and Lo-Cap slave sensor nodes, with a distributed intelligence.

In this context, hybrid WASNs [5] may play a significant role in the large-scale deployment of this kind of noise monitoring networks both in terms of cost and extent of coverage. These networks combine high-capacity (Hi-Cap) nodes with cheaper low-capacity (Lo-Cap) nodes, which operate as masters and slaves in the network, respectively (see Figure 1). This architecture allows sensing places where the power supply cannot easily be provided by means of solar panels or other alternative energy sources that supply the Lo-Cap sensors. Both nodes typically compute the A-weighted equivalent noise level ($L_{Aeq}$) of the monitored acoustic environment [17], being the Hi-Cap nodes also responsible for data communications and any other type of complex processes, e.g., acoustic signal processing, recordings, etc. In [11], the authors present the design of an acoustic sensor network based on this approach, with basic nodes using a low power μController (μC). RUMEUR network [18] is also an hybrid WASN, including both high-accuracy equipment for critical places, combined with less-precise measuring equipment in other locations whose purpose is only updating the noise map in terms of $L_{Aeq}$.

With the same focus, the DYNAMAP LIFE project is aimed at developing a dynamic road traffic noise mapping system to represent the acoustic impact of road infrastructures in real-time in two Italian pilot areas: a suburban environment in Rome and an urban environment in Milan [19]. To do so, the project envisions a hybrid low-cost WASN including both Hi-Cap and Lo-Cap salve sensors, which will be located in places with limited power supply. The DYNAMAP project takes into account a noise monitoring challenge not faced by the aforementioned projects as it is only focused on one specific noise source: road traffic noise. Hence, it considers the inclusion of an Anomalous Noise Event Detector (ANED) [15] to provide a reliable picture of the actual RTN by minimizing the influence of other anomalous noise events [20]. Up to now, the ANED algorithm has been designed as a two-class classifier (ANE vs. RTN) using Mel Frequency Cepstral Coefficients (MFCC) [21] as acoustic parametrization and supervised machine learning classification to run in real-time on the Hi-Cap sensors of the hybrid WASN, showing promising results on real-life data. However, the Hi-Cap ANED algorithm [15] cannot be implemented as originally designed in the slave Lo-Cap sensors due to the computational resources it demands. Nevertheless, it would be desirable to include an adapted version of the algorithm to run on the Lo-Cap sensors in order to provide an homogeneous picture of the RTN, thus, allowing the hybrid WASN to discard ANE from the $L_{Aeq}$ computation also in those locations where the slave sensors will be placed.

This paper describes the adaptation of the Hi-Cap ANED algorithm to run in real-time on Lo-Cap sensors (ANED Lo-Cap), analyzing its viability in terms of computational load and classification accuracy. The ANED Lo-Cap algorithm bases on the same principle of the original ANED version to discriminate between ANE and RTN to obtain consistent results across the nodes of the hybrid WASN, but adapted to fit the computational capacity of a Lo-Cap sensor. For this purpose, the approach follows a threshold-based binary classification scheme that considers the spectral energy differences between ANE and RTN [22] for the most discriminatory Mel-based Frequency Subbands (MFS). The experiments are conducted considering 9h and 8 min of real-life acoustic data from the two pilot areas (urban and suburban) of the DYNAMAP project.

This paper is structured as follows. Section 2 reviews the main works about wireless acoustic sensor networks and acoustic event detection in urban areas. In Section 3, we describe the theoretical foundations to design the ANED Lo-Cap proposal. Section 4 evaluates the feasibility of the frequency range selection of the ANED Lo-Cap using a real-life database of urban and suburban acoustic data in the framework of the DYNAMAP project. Section 5 evaluates the computational load necessary to implement the ANED Lo-Cap, considering several commercial hardware platforms. Section 6 discusses the viability of the proposal, considering several open questions foreseen from the obtained results, which should be tackled in future works, and Section 7 details the final conclusions of this paper.

## 2. Related Work

In this section, we describe representative approaches developed to automatically measure the noise levels of the cities in order to tailor noise maps [5]. The first part reviews the environmental sound classification approaches that can be found in the literature to face acoustic event detection in urban environments, with special focus on those works considering real-life operating scenarios. The second part reviews several noise monitoring projects and their platform design and hardware.

### 2.1. Acoustic Event Detection in Urban Environments

In this section, we review the most significant works focused on Acoustic Event Detection in urban environments based on the one-class novelty detection approach due to their similarity with the problem our proposal faces. Besides that, some works focused on traffic noise and urban soundscapes based on multiclass classification are also included.

Ntalampiras et al. describe a probabilistic novelty detection approach for acoustic surveillance under *pseudo*-real-life conditions [12]. The study includes normal and abnormal (or anomalous) audio events such as screams, shouting or pleading for help, which are collected in real-life outdoor public security scenarios. The acoustic data is parametrized every 30 ms using a multidomain feature vector including different audio descriptors, such as Mel Frequency Cepstral Coefficients (MFCC), MPEG-7 low-level descriptors (LLD), Intonation and Teager Energy Operator, and Perceptual Wavelet Packets (PWP). The parametrized audio frames are fed into different probabilistic classifiers based on Gaussian Mixture Models (GMM) and Hidden Markov Models (HMM), following a one-class classification (OCC) approach based only on the majority class (i.e., no information from the anomalous events is provided to the classifier): GMM clustering, universal GMM (UGMM), and universal HMM. It is worth mentioning that the hazardous situations are simulated with professional actors, thus, the gathered data cannot be strictly considered as collected in actual real-life conditions.

Later, in [23], Ntalampiras introduced an approach for acoustic surveillance in urban traffic environments following a multi-class AED approach. In that research, the AED is based on a two-stage HMM-based classification system and a window length of 30 ms, which analyzes a multidomain feature set, including again MFCC, LLD and PWP to consider the time, frequency and wavelet domains. The work includes a database composed of nine audio classes: car, motorcycle, aircraft, crowd, thunder, wind, train, horn and crash. The samples are obtained from several professional sound effect collections assuring high quality without being affected by background noise. Moreover, the detection of crash incidents is also studied by merging those sound events with the rest of classes at specific SNRs (i.e., 0, 5 and 15 dB). Thus, experimental configuration of the proposal is far from that obtained in real-life recording conditions since the events of interest are artificially mixed with background noise.

Aurino et al. apply OCC based on Support Vector Machines (SVM) to detect anomalous audio events within an automatic surveillance framework [24]. Specifically, the work is focused on the recognition of three types of burst-like acoustic anomalies: gun-shots, broken glasses or screams, which are defined and used to train one OCC-SVM per class a priori. The system follows a two-stage classification scheme by classifying the short audio segments (of a window length of 200 ms in the experiments) at the first level through an ensemble of OCCs. Then it aggregates these classification outputs into intervals of 1 s, which are subsequently reclassified using a majority voting strategy. The audio events are parametrized using a set of typical audio features such as MFFCs, Fast Fourier Transform (FFT), or Zero Crossing Rate (ZCR), to name a few. Again, it is to note that the events of interest are artificially mixed with background noise acquired in indoor and outdoor environments.

In [25], a real-life urban sound dataset named UrbanSound and its compact UrbanSound8k version are introduced. The dataset is composed of 10 low-level classes organized according to the following taxonomy: construction (drilling and jack hammer), mechanical (air conditioner and engine idling), traffic (car horn), community (dog bark, children play and street music) and emergency (gun shot and siren), whose events are artificially mixed with the background noise considering different SNR values and using a classification window length of 23.2 ms. The audio data are differentiated between foreground and background, depending on their acoustic salience. In order to study the characteristics of both datasets, an AED is also implemented and tested following a 10-fold cross-validation scheme. The AED is based on MFCCs together with other statistically derived features, and some classifiers provided by the Weka data mining platform. Recently, in [14] the authors have presented an AED following a local and global features aggregated based on a mixture of experts model, which is also tested using the UrbanSound8k dataset.

Foggia et al. have recently presented an adapted approach of their AED, focused on the detection of specific anomalous noise events, such as screams, glass breaking or gunshots [26] to detect abnormal sounds within urban traffic noise, such as tire skidding and car crashes using a low-cost hardware platform for urban surveillance [27]. A bag-of-words of sounds representation is used to perform AED after training a pool of SVM-based classifiers that consider different feature extraction techniques with a window time frame of 32 ms. The authors compare the results over both real-life and synthetic acoustic dataset, and the system shows that the MFCC plus the proposed classification approach presents the best performing configuration according to the ROC curves.

In [16] the authors design a classifier which goal is also to separate between target and interfering noise. The activity of the noise source is detected by means of a binary classifier discriminating between the target, which can be plant or aircraft noise, and the background, which can be traffic, wind, rain, thunder, etc. The algorithm is based on MFCC [21] 100 ms window length feature extraction with the classification using a supervised classifier (GMM and Artificial Neural Networks (ANN)), trained with an annotated real-life dataset.

Finally, and in the framework of the DYNAMAP project [19], a preliminary study on the Anomalous Noise Event Detector (ANED) for high capacity sensors was developed to differentiate between road traffic noise and anomalous noise events in both urban and suburban environments [15]. It was based on a two-class audio event classification approach to be implemented in a low-cost acoustic sensor of a WASN. The algorithm and the experiments were conducted following the project operating specifications, using raw real-life data collected from both urban and suburban environments by means of a recording campaign [28]. The results prove the viability of the two-class classification scheme to detect ANE, using a window of 30 ms, and MFCC to parameterize the audio, and GMM as probabilistic classifier, outperforming the OCC counterpart in both sampled urban and suburban environments.

### 2.2. Networks for Noise Monitoring

In this section, we review several representative approaches developed to automatically measure the noise levels in cities to tailor noise maps [5]. The analysis considers both the hardware approach and the platform design, with a special focus on hybrid network hardware proposals, where both Hi-Cap and Lo-Cap sensors are included in the network. The main goal of most of the networks is to measure and integrate the calculated $L_{Aeq}$ for a certain interval of time in a map. This analysis has divided the sensors into three categories: (i) high accuracy and low noise floor acoustic sensors, with high price of the sensor nodes in a WASN, (ii) low-cost nodes to design a WASN balancing the accuracy and the price and (iii) hybrid WASN, using both Hi-Cap and Lo-Cap nodes, balancing the cost and the efficiency of the data processing in the network.

The first category of WASN that can be found in the literature is built to achieve high accuracy and reliability, together with low noise floor. To that effect, most of their acoustic sensors are monitoring stations from Bruel and Kjaer [29] or Larson and Davis [30], which are equipped with IEC class 1 microphones. Those projects working with this kind of sensors are mainly focused on performing a detailed study of the acoustic environment of the city of interest. In [13,31], the FI-Sonic project based on the FIWARE platform is described; it consists of an acoustic sensor network based on ambisonics microphones, a multichannel acquisition card (from 2 to 128 GB), a network interface (with a Wi-Fi/3G modem) and a media server, its main processing unit, which runs the audio analyses. The collected information is used to create quasi-real-time dynamic noise and event maps, as well as to identify specific pre-trained sound sources for surveillance purposes. The FI-Sonic project is an example of the application of high accuracy WASNs to noise monitoring, but its pervasive deployment will require a very high investment. The problem associated with this first category of WASN is the price of the deployment of an entire network with several nodes, which may become prohibitive.

A second category of acoustic sensor networks is designed to balance the accuracy and the cost of the network. These WASNs are usually designed to be deployed in large networks, and the priorities in their design are not only price and accuracy, but also allow the possibility of processing real-time the acoustic signal in each node of the network. Some of the networks in this category are based on commercial sound level meters, such as the one used to monitor the traffic noise in Xiamen City (China) [32]. The designed WASN also considers ZigBee technology and GPRS communication, and all the nodes of the network use the same type of device. In the SENSEable project [33], a WASN was proposed to collect information about the acoustic environment of the city using low-cost acoustic sensors to study the relationship between public health, mobility and pollution through the analysis of citizens’ behaviour. Also within this second category, we can find several WASNs which can be deployed in a pervasive manner, such as the ones of the IDEA [6] and the MESSAGE [34] projects. They are based on a single board computer with low computational capacity, using low-cost sound cards. This hardware choice permits the deployment of large sensor networks due to the low economic cost of each node, besides allowing the collection of relevant environmental data from several critical locations in the city. In the IDEA project [35], a cloud-based platform is also developed by integrating an environmental sensor network with an informative web platform, which aims to measure noise and air quality pollution levels in urban areas in Belgium. Most of the aforementioned monitoring projects were only focused on measuring the $L_{Aeq}$ values, therefore, the nodes are only required to conduct their computation. When the application requires higher complexity in the processing of the acoustic signal, the computational capability of the nodes should be increased accordingly. Nevertheless, some of the acoustic sensor designs of this second category are developed ad-hoc for each project. Some projects even design the nodes of the WASN to conduct some signal processing over the acoustic raw data in the proper node of the WASN. In [36], the urban sound environment of New York City is monitored using a low-cost static acoustic sensing network.

The third category would be hybrid WASNs, which arise as a good trade-off between cost and required features of scalability, reliability and flexibility [5]. This typology of acoustic networks include both high capacity nodes, which are able to perform signal processing algorithms in real-time against the recorded data, and low capacity nodes, whose main goal is to compute the $L_{Aeq}$ value. This architecture is cheaper than the one only composed of high-capacity sensors, because the high capacity nodes are only deployed where needed, and it is scalable to wide zones at a lower cost. This hybrid architecturealso makes it possible to sense remote zones with no access to power supply by means of the use of a low-capacity sensor fed by solar panels. In [11], the authors present the design of an acoustic sensor network based on this approach. The hardware platform for a basic node is a low power μC whose main goal is to compute the $L_{Aeq}$ and transmit the collected $L_{Aeq}$ data periodically. The advanced nodes allow far more processing capabilities in comparison with the basic ones, since they use a small PC with a 2 GHz Intel Atom Processor running a Linux operating system. The advanced nodes can both store and process the acoustic data, which are designed to be flexible for the necessary signal processing analyses. Also in [37], the authors obtain the measurements of the RUMEUR project from sound level meters installed in a sensor network that pursues the understanding the measured signal, assess actions to mitigate noise and communicate the information about the soundscape in Ile-de-France. The RUMEUR hybrid networks [18] includes both high accuracy equipment for critical places, like airports, where the focus is to obtain detailed acoustic information due to the intense noise environment, together with less precise measuring equipment in other locations aimed at only updating of the noise map with the corresponding $L_{Aeq}$ level. Achieving a good trade-off between cost and accuracy is also the core idea of the WASN design in the DYNAMAP project [19,38]. This project is aimed at the deployment of a low-cost WASN in two pilot areas in Italy, located in Rome [39] and Milan [40], so as to evaluate the noise impact of road infrastructures in suburban and urban areas, respectively. The sensors designed for the DYNAMAP project are low-cost and use class 2 MEM microphones. This hybrid WASN will deploy two types of sensors: (i) high capacity ARM-based sensors, allowing signal processing techniques to analyze and process the acoustic signals in each node [38], and (ii) low capacity μController (μC)-based sensors, with less computational capabilities and fed by solar panels, but more flexible in terms of sensor positioning, maximizing the coverage of the network.

## 3. ANED Lo-Cap: An Anomalous Noise Event Detector for Low-Capacity Acoustic Sensors

Section 2.1 describes several AED algorithms developed to identify noise sources. However, the particular requirements of the DYNAMAP project pose the need of designing and implementing an algorithm capable of detecting ANE within RTN in real-time. The main goal of the Anomalous Noise Event Detector described in [15] is identifying ANE dynamically to allow a reliable representation of the A-weighted equivalent noise level of RTN (see Figure 1) in outdoor acoustic environments. This section describes our proposal for adapting the ANED Hi-Cap [15] to run real-time in Lo-Cap sensors, following a similar classification principle to discriminate between ANE and RTN in order to obtain consistent results across the hybrid WASN.

In this section, we firstly overview a general description of the method; then, we describe the acoustic signal parametrization used to characterize the RTN and ANE acoustic signals. We finally detail the first stage of the optimization process of the ANED Lo-Cap by means of studying the spectral energy distribution of both ANE and RTN acoustic categories.

### 3.1. General Description

The ANED Lo-Cap algorithm proposal is mainly focused on the reduction of the computational complexity of the ANED algorithm [15], while maximizing its capability of identifying ANE to discard them from the RTN noise level computation. In order to implement the algorithm in an affordable Lo-Cap platform with a μC core, a change of paradigm on the AED is mandatory, both in terms of feature extraction and classification, to assume the severe decrease of the computational load of the Lo-Cap platform with respect to the Hi-Cap counterpart. As studied in [22], the distribution of the energy per subband show signs of separability depending on the frequency between ANE and RTN, and the proposal presented in this piece of research deepens the analysis in this direction.

**Figure 2:**
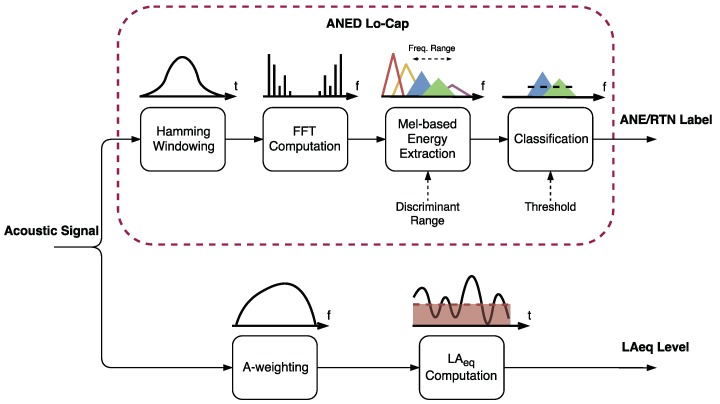
Block diagram of the acoustic signal processing within the Lo-Cap acoustic sensor. The upper part details the block diagram of the ANED Lo-Cap, with a binary label as an output, and the lower branch details the evaluation of the $L_{Aeq}$ of the measured acoustic signal.

In Figure 2, the block diagram of the acoustic signal processing within the Lo-Cap acoustic sensor is depicted. It includes both the ANED Lo-Cap and the computation of the $L_{Aeq}$. The upper part of the Figure, corresponding to the ANED Lo-Cap, details that the algorithm starts windowing the acoustic signal registered in the sensor before conducting the Fast Fourier Transform (FFT) [41] on each input acoustic frame. After that, specific Mel frequency subbands [21] of a pre-studied frequency range outputs are computed as a simple frequency-based scalar product between the squared FFT module and the MFS filter. Next, the spectral energy should be computed from the discriminant range in frequency is compared to a pre-calculated threshold (obtained by means of an optimization process), leading to the classification of each input frame as RTN or ANE accordingly. In parallel, the $L_{Aeq}$ is obtained through an A-weighted filter applied to the input acoustic signal and subsequently computing the equivalent energy level in dB.

### 3.2. Acoustic Signal Parameterization

The ANED designed for the Hi-Cap acoustic sensors [15] used MFCC [21] to parametrize the input acoustic data. With the same basis, the ANED Lo-Cap uses a simplified frequency-based representation based on a Mel filter-bank analysis in order to maintain the homogeneity of the signal analysis in all the nodes of the hybrid WASN while reducing the computational cost of the signal parametrization. The use of the Mel-based frequency scaling simulates the way the human ear works, having a higher resolution in low than in high frequencies [21], which is a good approach for the spectral distribution of the sounds under study [42,43]. The last stage of the MFCC computation which is basically used for dimensional reduction of the obtained feature vector is here omitted, thus, the classifier uses directly the output energies of the pre-selected MFS.

### 3.3. Optimization of the ANED Lo-Cap Configuration

In this section, we describe the process to obtain the frequency region that maximizes the discrimination between ANE and RTN and also the methodology followed to set the decision threshold. Firstly, the MFS distribution is analyzed to obtain the most suitable frequency range in terms of class separability. This separability is evaluated by means of the results of probability of error for each MFS studied subband, which will be detailed in Section 3.3.1.

**Figure 3:**
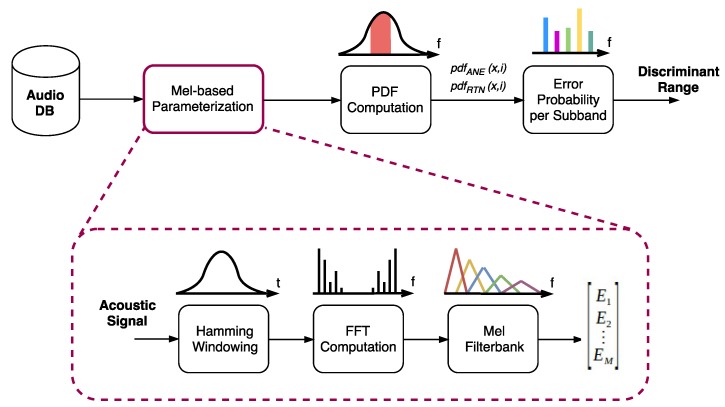
Block diagram of the process conducted to select a frequency range with minimum probability of error of classification.

The block diagram of the optimization process is shown in Figure 3. The first stage comprises the MFS parameterization of the entire acoustic dataset, which comprises the windowing of the acoustic signal using a Hamming window [44] of length $T_{w}$, the FFT computation of each acoustic frame and the calculation of the output energies ($E_{i},1\leq i\leq M$) of a *M*-Mel-based filter bank using a simple scalar product in the frequency domain. Next, two Probability Density Functions (PDFs) of the logarithmic signal level *x* (in dB) at each *i* frequency subband are computed, taking into consideration all the signal frames of the dataset that belong to each acoustic class: one for the ANE class, $pdf_{ANE}\left(x,i\right)$, and another for the RTN class, $pdf_{RTN}\left(x,i\right)$. These two PDFs are the basis to obtain the one-band threshold-based linear discriminators for classification purposes, through which an error probability function can be derived in terms of each of the *M* frequency subbands, $p_{e}\left(i\right)$. This leads us to obtain the most suitable frequency range to discriminate, and thus, classify an input acoustic frame as ANE or RTN.

In Section 3.3.1, the procedure used for computing an error probability function per subband ($p_{e}\left(i\right)$) is elaborated, with the goal of obtaining the subbands that improve the classification. Then, Section 3.3.2 explains the criteria used to select the best frequency range to use for the ANED Lo-Cap based on the evaluation of this error probability function.

#### 3.3.1. Computation of the Probability Error Function

Given the pair of PDF distributions, $pdf_{ANE}\left(x,i\right)$ and $pdf_{RTN}\left(x,i\right)$, which describe the logarithmic energy density distribution of ANE and RTN classes, respectively, in terms of the frequency subband *i* (see Table 1), two types of total error functions are computed based on two hypotheses:

**Hypothesis 1** **(H1).** *class ANE has signal energy levels that are above those of class RTN. For this hypothesis, the total error function is defined as $p_{e}^{ANE>RTN}\left(i\right).$*

**Hypothesis 2** **(H2).** *class RTN has signal energy levels that are above those of class ANE. For this hypothesis, the total error function is defined as $p_{e}^{ANE<RTN}\left(i\right)$*.

Both error functions also depend on the energy level threshold γ that will be used to discriminate between the two classes, which are defined as follows:

(1) $$\begin{array}{c} p_{e}^{ANE>RTN}\left(i,\gamma \right)=P_{ANE}\int_{-x_{min}}^{\gamma }{pdf}_{ANE}\left(x,i\right)dx+P_{RTN}\int_{\gamma }^{x_{max}}{pdf}_{RTN}\left(x,i\right)dx\;\\ p_{e}^{ANE<RTN}\left(i,\gamma \right)=P_{RTN}\int_{-x_{min}}^{\gamma }{pdf}_{RTN}\left(x,i\right)dx+P_{ANE}\int_{\gamma }^{x_{max}}{pdf}_{ANE}\left(x,i\right)dx \end{array}$$

where $P_{ANE}$ and $P_{RTN}$ are the *a priori* probabilities of class ANE and class RTN, respectively, and $x_{min}$ and $x_{max}$ are the minimum and maximum observed signal energy level at frequency subband *i*, respectively.

For each *i*th-frequency band, the optimum energy threshold γ is defined as the one attaining the minimum value of the corresponding error function for each hypothesis ($\gamma_{opt}^{H1}\left(i\right)$ and $\gamma_{opt}^{H2}\left(i\right)$). As it can be observed in Equation (1), the total error functions are computed as the sum of two type of errors (*type I*: classifying ANE when class RTN, *type II*: classifying RTN when class ANE), and their evaluation will differ depending on the way the PDF is evaluated. For each *i*th-frequency band, the minimum error is computed for both error functions substituting its corresponding optimum threshold. The final decision is determined by considering the most feasible hypothesis, whether to accept H1 or H2. Thus, the final error probability function $p_{e}\left(i\right)$ is defined as the minimum of both error functions ($p_{e}^{ANE>RTN}\left(i,\gamma \right)$ and $p_{e}^{ANE<RTN}\left(i,\gamma \right)$) for each frequency band (see Equation (2)), and the optimum decision threshold function (see Equation (3)) is computed using the corresponding optimum threshold for each frequency subband index *i*. 

(2) $$p_{e}\left(i\right)=\left\{\begin{array}{cc} p_{e}^{ANE<RTN}\left(i,\gamma_{opt}^{H2}\right) & \mathrm{if}\;p_{e}^{ANE>RTN}\left(i,\gamma_{opt}^{H1}\right))>p_{e}^{ANE<RTN}\left(i,\gamma_{opt}^{H2}\right)\;\\ p_{e}^{ANE>RTN}\left(i,\gamma_{opt}^{H1}\right)) & \mathrm{otherwise} \end{array}\right.1\leq i\leq M$$

(3) $$\gamma_{opt}\left(i\right)=\left\{\begin{array}{cc} \gamma_{opt}^{H2}\left(i\right) & \mathrm{if}\;p_{e}^{ANE>RTN}\left(i,\gamma_{opt}^{H1}\right))>p_{e}^{ANE<RTN}\left(i,\gamma_{opt}^{H2}\right)\;\\ \gamma_{opt}^{H1}\left(i\right) & \mathrm{otherwise} \end{array}\right.1\leq i\leq M$$

#### 3.3.2. Selection of Frequency Range

Once the error probability function $p_{e}\left(i\right)$ has been obtained for a given acoustic dataset (see Equation (2)), its evaluation can be used to define a suitable frequency range use in the classification in terms of ANE and RTN discrimination capabilities [22] (see the last module in Figure 3). This selection of frequency range is defined following the next simple rule: including those MFS subbands for which the error probability function is less or equal than a certain probability of error threshold. The computation of the decision threshold of the last stage of the ANED Lo-Cap (see Figure 2) follows a similar procedure of that used for the one-band linear discriminators [45], and should be conducted ad-hoc after the calculus of the probability of error.

## 4. Experimental Section

This section details the experiments conducted to validate the viability of the ANED Lo-Cap algorithm in terms of probability of error and computational requirements within the framework of the DYNAMAP project. The tests are conducted using two real-life acoustic datasets from two different outdoor acoustic environments: urban and suburban. The probability density functions are obtained for both scenarios, and the most discriminative frequency subband ranges are pointed out.

### 4.1. Acoustic Database

In this section, the acoustic database analyzed to validate the low-capacity version of the ANED is briefly described in [46]. This database includes two different acoustic scenarios regarding road traffic noise monitoring: a suburban dataset including a major road (along the A90 highway surrounding Rome); and an urban dataset (within the district 9 of Milan), which comprises different types of roads, as well as several traffic density conditions. The two acoustic datasets were obtained using low-cost acoustic sensors coupled to a ZOOM H4n digital recorder (ZOOM, Hauppauge, NY, USA), and considering a 48 kHz sampling rate with 24 bits/sample. As a result of the four-day recording campaign between the suburban and urban scenarios, a total of 9 hours and 8 minutes of audio were collected.

Subsequently, a manual labelling process was conducted on the collection of audios gathered from both real-life scenarios, which entailed exhaustive listening by experts and the subjective classification of the acoustic data between RTN (differentiating between road traffic and background city noise when it was not perceived the presence of vehicles) and ANE (considering up to 19 labels to describe different noise sources, such as vehicle horns, airplanes, people talking, sirens, music in the street, noise coming from train or tramways, etc.).

ANEs were also labeled in terms of their acoustic salience with respect to the surrounding noise by firstly obtaining an estimation of the Signal-to-Noise Ratio (SNR in dB), following the computation approach explained in [20]. This provides information which is valuable when it comes to considering or rejecting ANEs with low SNR (e.g., SNR≤0) when training the ANED as stated in [20], since it does not have a significant impact on the final $L_{Aeq}$ computation.

In this work, the input acoustic signal is segmented into 30 ms frames using a Hamming window with 50% of overlap [44]. The input acoustic frame is transformed to the frequency domain through the FFT [41] after being sampled at 48 kHz. Then a filter-bank of M=48 Mel filters is applied to the spectrum of the signal’s square module, covering the entire human audio range from 20 Hz to 20 kHz [47], obtaining the corresponding MFS. The computational load of the signal processing is reduced when applying the filter-bank in the frequency-domain compared to the alternative convolution in the time-domain [48]. The central frequency of each of the 48 MFS used for the ANED Lo-Cap version—which follow a logarithmic distribution—are shown in Table 1.

### 4.2. 2D-PDF Subband Analysis and Selection

This section details the computation of the PDFs in the framework of the DYNAMAP project, considering the two real-life acoustic datasets from the urban and suburban environments, respectively. These PDFs will permit us to design for each Mel-frequency band a one-band threshold-based linear discriminator for binary classification purposes, and an error probability function will be derived for all subbands in order to observe which frequency range is the most suitable for each environment.

#### 4.2.1. Computation of the PDFs for Both Scenarios

In this section, the resultant energy level PDFs are obtained for both signal classes, RTN and ANE, by considering the real-life data from the two pilot site recordings. The two datasets, already parameterized through the MFS, have been used as the main input data for the computation. Energy level PDFs have been estimated using simple histograms for each of the M=48 frequency bands of the MFS [15], obtaining two-dimensional functions (2D-PDF). For each spectral subband, the PDF is computed using a minimum of 200 samples per class (for accuracy reasons) within the range of observed values of the MFS energy level outputs. For this study, only those ANEs with SNR≥0 have been considered in order to minimize the potential confusions between RTN and ANE that could minimize the events separation probability. More details about the evaluation of the SNR in all the ANE in the dataset can be found in [20].

**Figure 4:**
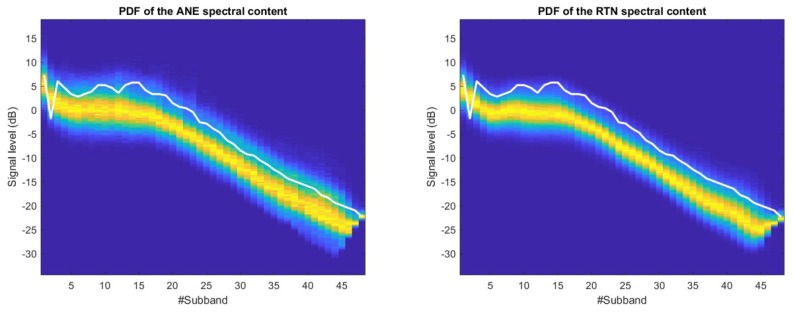
2D-PDF of the ANE (**left**) and the RTN (**right**) of the suburban dataset. The frequency subband index *i* corresponding to the MFS is labelled in the x-axis (the reader can find the corresponding frequency in Table 1), while the corresponding logarithmic signal level at each subband is depicted in the y-axis in dBs. The Figures colormap is blue for lower probabilities while tends to warm colors (with the maximum in red) for higher probabilities. The solid white line represents the optimum decision threshold for the one-band linear discriminant classifier at each frequency bin.

In Figure 4, the 2D-PDF matrices corresponding to the MFS distributions of ANE and RTN in the suburban scenario are depicted. For illustrative purposes, all the 2D-PDF visualizations have been scaled at each frequency subband dividing each PDF by its maximum value in order to obtain a more comprehensible plot, so the absolute value of probability is normalized. Also, the optimum decision threshold (see Equation (3)) is plot as a solid white line in both axes across the frequency index.

It is worth noting the high similarity of the 2D-PDFs of both ANE and RTN plots for the suburban acoustic environment, showing also an important overlap between them. However, the ANE class presents a slightly wider variance than the RTN class along the signal level axis for most of the MFS (i.e., an increase of about 10 % in the variance values for the ANE class is found with regard the RTN variance). However, a decrease of this signal level dispersion of both distributions can be observed along the frequency subband indexes from **23** to **27**. Then, within those subbands the overlap of both signal level distributions (ANE and RTN) are lower, being the ANE class the one that attains higher signal levels (e.g., its 2D-PDF maximum probabilities, represented in yellow color, are placed closer to the optimum decision threshold). Another subband to analyze is number **2**, which presents a lower value than subbands **1** and **3** in the analysis of both ANE and RTN. Subband **2** corresponds to a central frequency of 153 Hz, which is one of the most common frequency ranges of road traffic noise [42,43], and yet in this location the curve of the threshold adapts to the best possible discrimination between ANE and RTN following Equations (2) and (3).

**Figure 5:**
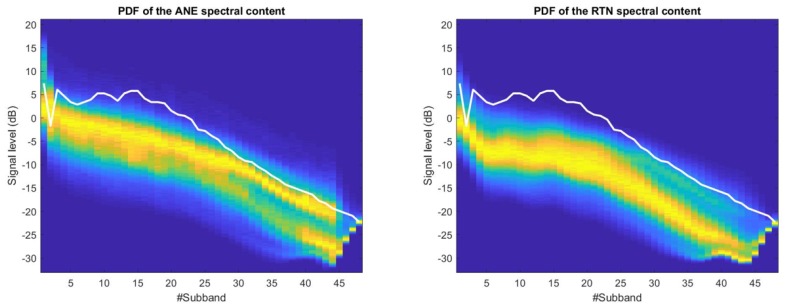
2D-PDF of the ANE (**left**) and the RTN (**right**) of the urban dataset. The frequency subband index *i* corresponding to the MFS is labelled in the x-axis (the reader can find the corresponding frequency in Table 1), while the corresponding logarithmic signal level at each subband is depicted in the y-axis in dBs. The Figures colormap is blue for lower probabilities while tends to warm colors (with the maximum in red) for higher probabilities. The solid white line draws the optimum decision threshold for the one-band linear discriminant classifier at each frequency bin.

Figure 5 shows the 2D-PDF matrices corresponding to the urban recordings. This acoustic scenario presents clearer differences between the 2D-PDF plots of the ANE and RTN classes compared to the suburban environment. Moreover, evidences of two clear spectral patterns can be observed in the ANE 2D-PDF: the first one located at high energy levels, and the second one that entails lower energy levels, especially from subband **27** and higher frequencies. In addition, differences between ANE and RTN are more evident in the lower frequency band (e.g., Mel subbands **5** and **6**). Subband **2** in the urban environment follows the same performance as in the suburban, and the threshold is set to a lower value than the neighbour subbands in order to minimize the probability of error.

#### 4.2.2. Subband Error Probability Calculation

After the 2D-PDF computation for each use case and type of signal, a threshold optimization is performed for each and every MFS, being M=48 (see Section 3.3). The calculated threshold can be observed as a white solid line in both Figure 4 and Figure 5. This threshold enables us to evaluate the probability of error for each of the MFS, and so conclude in which frequency region the separability between ANE and RTN is optimum to maximize the accuracy of the subsequent classification.

**Figure 6:**
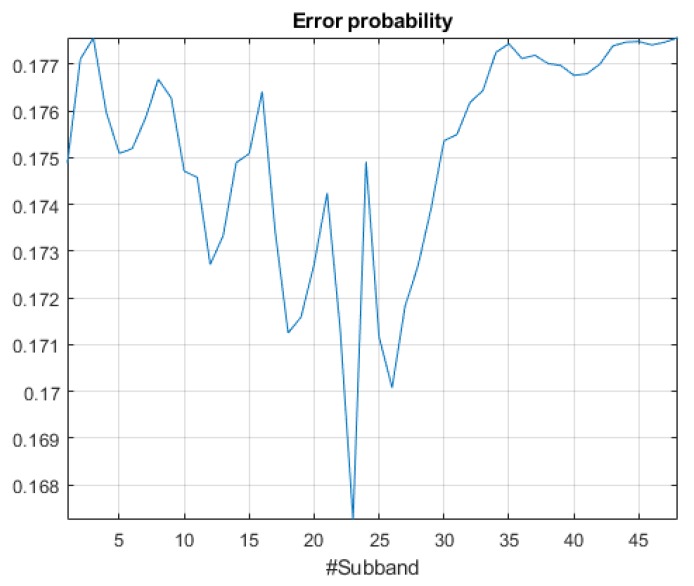
Error probability analysis for suburban recordings data.

In Figure 4, we can observe a high overlap between the two PDF functions that lead to a hardly stable threshold function, showing a smooth behaviour across the M=48 frequency subbands. Figure 6 shows the plot of the final error probability function defined in Equation (2) for the suburban dataset. The absolute minimum error probability is achieved in band **23**, being 0.1673. The error probability exhibits also a frequency region with a second local minimum value of 0.17, around subband **26**, and another third local minimum around subband **18**. This leads us to conclude that in the suburban environment, the most suitable frequency region to take into account in terms of separability can be found within the mid-frequency range.

**Figure 7:**
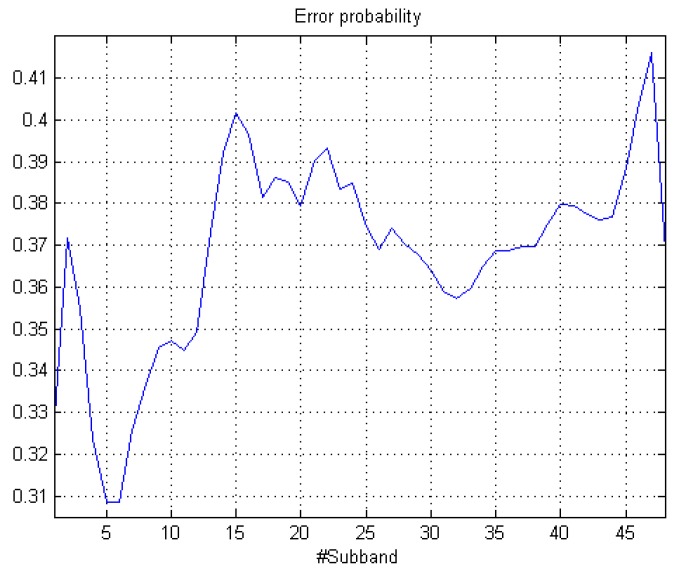
Error probability analysis for urban recordings data.

Figure 5 shows the results of the threshold for the urban acoustic database plotted in solid white line. Figure 7 shows that the minimum error probabilities are obtained for subbands **5** and **6**, presenting the minimum value ($p_{e}^{ANE>RTN}\left(x\right)=0.31$ in the 6th subband, see Equation (1) for more details). Moreover, subband **1** also yields a third local minimum of the computed error probability. This leads us to conclude that in the urban environment, the low-frequency region is the most suitable to discriminate between ANE and RTN.

## 5. Operations Cost Analysis of the ANED Lo-Cap

In this section, we evaluate the operations cost of the ANED Lo-Cap to determine the minimum features of the hardware platform required. First, the audio acquisition restrictions are detailed and, after that, the audio processing computational costs are calculated for the three main stages of the ANED Lo-Cap proposal: windowing, Fast Fourier Transform (FFT) [41] computation and subband filtering. After the overall energy that includes the optimized subbands is obtained, a comparison with a decision threshold is conducted to finally classify between RTN and ANE.

### 5.1. Audio Acquisition

The computational cost of the audio acquisition cannot be simply calculated as the number of floating point operations, since it depends on the architecture of the platform where it has to perform real-time. However, the μC or the processor will require a certain time dedication to the audio data reading process. If the ANED Lo-Cap is implemented on an ad-hoc hardware solution, a buffer can be used to store the audio data, and the data from the buffer would be input to the processing unit. However, if the data should be retrieved directly from the sensor, i.e., using an Analog-to-Digital Converter (ADC), it will require the processor to read periodically the data at the desired sampling rate. Therefore, the time cost of the audio acquisition system will increase.

In the best case, the audio acquisition system should be able to sample the input data at the same rate as the audio sampling frequency, in this case 48 kHz. However, a lower sampling rate could be considered if we assume that the ANED Lo-Cap will only detect low-frequency ANEs and an anti-aliasing filter is implemented before the data acquisition.

### 5.2. Acoustic Signal Processing

In order to extract the energy for each subband, three steps should be followed. First, a time windowing of *N* points is applied in order to extract the audio samples of the input frame. After that, the FFT is computed for the desired number of points, in this case, the same as the number of samples, i.e., *N*. Finally, the subband filter of *C* coefficients is applied to the spectrum of the frame to calculate the energy of the subbands of interest. Table 2 synthesizes the minimum number of operations required to implement the ANED Lo-Cap using the triple stage procedure detailed in this section.

- **Windowing:** In order to analyze short frames of audio, a window function should be applied to the input signal in order to reduce the spectral leakage due to higher frequencies. In this implementation, the hamming window is used [44]. The computational cost associated to any windowing process depends on the number of samples of the analyzed frame if the window function is computed and stored in advance. The ANED Lo-Cap proposal uses time frames 30 ms long, thus, if the sampling frequency is 48 kHz, the window will be 1440 samples long.
- **FFT Computation:** The FFT is one of the most popular algorithms that computes the DFT (Discrete Fourier Transform) of a sequence reducing its complexity by factorizing the DFT matrix. The most used algorithm is the Cooley-Turkey [41], that breaks the down the DFT of *N* points into smaller ones, typically dividing it in two pieces of N/2 at each step. The computational cost of the FFT may vary depending on *N* (the number of points of the FFT) and on the methodology of implementing the algorithm over a certain hardware platform and its optimization. In our case, the FFT shall be of minimum 1440 points and maybe of 2048 after adding zero-padding if the used algorithm requires a power-of-2 size.
- **Sub-band Filtering:** After the FFT is computed, a triangular-shaped filter is applied to a determined subband. The computational cost of obtaining each filtered subband depends on the number of coefficients (*C*) of the filter, which, in its turn, depends on the sampling frequency and the number of points of the FFT. The filter is used to obtain the energy of the subband, hence, the computational cost should consider the point-to-point multiplication of the vector and the filter and the posterior integration of the resulting vector. In order to reduce the computational cost, the filter may be designed in advance considering the sampling frequency and the number of points of the FFT. After that, only a product for each bin followed by a sum of all resulting outputs will be needed. The number of operations can be reduced if the filter is only employed in the concerning subbands and all other frequencies are omitted. In our case, two Mel subbands shall be implemented as it is the combination with a lower probability of error.

Depending on the efficiency of the algorithm implementation over every type of hardware platform the total computing time will tend to this evaluation or will be higher, if the implementation is not optimum. It could even be lower if the algorithm is optimized for a determined platform and several operations were omitted or replaced by hardware.

### 5.3. Commercial Board Comparison

In this section, we detail the overview of several commercial platforms that could be used to implement the ANED Lo-Cap. We evaluate their features and their computational capacity to host the performance of the ANED Lo-Cap. Nevertheless, in the framework of the DYNAMAP project an adhoc low-capacity platform is being developed to host the slave nodes of the WASN in both pilots.

The choice of a commercial board as the hardware platform capable to run the ANED Lo-Cap is delimited by its audio acquisition and processing requirements. The typical commercial boards can be classified according to the core processing system, which could be a μC, a μProcessor (μP) or a Field-Programmable Gate Array (FPGA).

The economic μC-based boards present many drawbacks, but they could serve as a first approximation to the ANED Lo-Cap. As an example, an Arduino is used for this viability study. According to the tests conducted in [49], the analog read function in Arduino in a loop takes 39 μs, making it impossible to sample at more than 25 kHz in the ideal scenario and using a full dedication of the controller. In this case, the FFT computation in real-time would be impossible. However, a fine tuning of the board registers could improve the reading speed to a 4 μs and a higher sampling rate could be obtained. Still, in order to work real-time, a cooperative implementation of the FFT and the ADC read should be coded and a deep understanding of the internal registers should be achieved by the developer. The implementation, if possible, would have a double restriction, i.e., the sampling frequency and the number of points of the FFT.

A second approximation could be conducted with ARM-based boards, where ARM stands for Advanced RISC Machine. In this case, the processing capacity allows the FFT computation and some boards, e.g., Raspberry Pi, even allow the acceleration of the FFT using the Graphics Processing Unit (GPU) [50], facilitating the computation and freeing the Central Processing Unit (CPU). However, not all these boards implement an ADC, complicating the task of acquiring the audio. The Raspberry Pi, for example, does not include an internal ADC and an ad-hoc system must be included. Nevertheless, other ARM-based μC boards offered by NXP [51] and STM [52], have ADCs that sample over 1 MSPS, enough for audio acquisition.

A third approach could be using FPGA-based platforms, which entail a completely different architecture and programming paradigm. The processing capacity of the FPGAs is limited basically by the space and it is suitable for parallel executions but it is not optimal when programming complex sequential tasks. In our case, the FFT computation can be easily implemented by using the manufacturer libraries, as the offered by Xilinx (San Jose, CA, USA) [53]. Also, several platforms offer high-speed internal ADCs, as some Xilinx boards or the Arty A7 [54], also built based on a Xilinx FPGA.

From the detailed approaches, the most economic one does not fulfil the basic requirements (e.g., Arduino), a 48 kHz sampling rate with real-time subband energy extraction. However, both microprocessor and FPGA-based boards are able to carry the needed operations in real time. The final decision could be supported by the information in Table 3, which lists the basic price of each commercial hardware platform considered; the first platform that can satisfy restrictions is the Raspberry Pi Model A+, and the price is $20, which is the same price as the Arduino. It includes ad-hoc audio input, which would allow to receive the audio in real-time with no need to constantly stop the processor to read new data.

The ANED Lo-Cap applies only a reduced number of Mel filters and does not compute the Discrete Cosine Transform (DCT) and the GMM of 13 coefficients. Therefore, the computational load is reduced in a factor of around 6 in comparison to the ANED Hi-Cap. In this case, the authors have estimated that the Hi-Cap version of the ANED could no run in this platform as the computational load is six times higher and it requires to have remote access, storage and other monitoring tools to control all the connected Lo-Cap slave nodes. The other platforms, although they could be considered to be low-cost, have higher starting prices, which considering the deployment of a network with a lot of nodes, may alter substantially the global budget.

## 6. Discussion

In this section, we discuss whether the use of an ANED Lo-Cap in the low-capacity nodes of an hybrid WASN is feasible and reach the real-time hardware and accuracy requirements by considering three different aspects. The first one deals with the accuracy results in terms of the Macro-averaged F1 measure; in this case, the evaluation takes into account full-band analysis and random threshold selection as reference and the ANED Hi-Cap counterpart applied to the same acoustic data. The second one deals with the homogeneity of the network performance. Finally, the third one analyses the computational load of the proposed algorithm and its real-time performance in the studied low-cost hardware platforms.

### 6.1. Classification Accuracy of ANED Lo-Cap vs. ANED Hi-Cap

This article focuses on the design of an ANED Lo-Cap for low-cost platforms and the evaluation of its viability using real-life data, but leaving for future works the development of, exhaustive tests in a real-operation environment. Nevertheless, we have conducted some preliminary analyses to establish a performance baseline for the two studied scenarios, following a 4-fold cross validation scheme.

The results in the suburban scenario considering all the Mel-based subbands (M=48) yield a Macro-averaged F1 measure [55] of 51.6% (with a $\sigma_{F1}=0.37$). Although this accuracy is quite low, it is to note that it is indeed higher than the one obtained using a non-optimized threshold, i.e., a threshold based on an uniform random selection within the range of the measured signal energies. In this case, the ANED Lo-Cap accuracy decreases up to 50.4%. If we compare the F1 values obtained by the ANED Lo-Cap and the ANED Hi-Cap [15] in this scenario, we can observe that the subband optimized ANED Lo-Cap is around 9% less accurate on average, but allowing a computational load decrease in an order of 6 times (see Section 5). Finally, we want to note that some preliminary tests considering subband selection show promising results, with an averaged increase of around 3% in the F1 measure with respect to the full-band ANED Lo-Cap.

The results in the urban scenario yield a Macro-averaged F1 measure of around 62% (with a $\sigma_{F1}=0.17$) considering the full Mel-based frequency range. The ANED Lo-Cap presents better results in the urban than in the suburban environment (showing an increase of more than 10% in terms of accuracy); a pattern already observed for the ANED Hi-Cap version [15].

In this scenario, the non-optimized threshold baseline classification system decreases 4.8% the averaged F1 values with respect to the Lo-Cap ANED optimized proposal for the full-band configuration. Therefore, considering this result and the one for the suburban environment, we can conclude that the threshold optimization is a significantly valuable process for the proposed algorithm. Moreover, comparing these results with the ones obtained by the ANED Hi-Cap in the urban environment—reaching 72.7%, the proposed ANED Lo-Cap is also around 10 % less accurate in terms of the F1 measure. Morever, the preliminary tests including some kind of frequency range selection show a relevant improvement with an averaged increase of 5% in the F1 measure. Nevertheless, we shall study in more detail these results for the optimal implementation of the ANED Lo-Cap in a real-life operating framework.

The conducted experiments have considered a 30 ms frame-based classification analysis. In [15], a higher decision level was also included to obtain a binary output every 1 second by means of a majority voting scheme. As a result, some of the noisy decisions of the ANED Hi-Cap were reduced, as the majority vote works as a low-pass filter of the frame-based decisions, thus, improving the F1 results in around 5 %. It is to note that the majority vote has not been yet implemented in the ANED Lo-Cap, being left for a second stage of the development of the proposed algorithm, as it is not critical in terms of computational load in comparison with the entire proposal.

These preliminary results obtained for both environments encourage us to, at least, keep working to improve the algorithm for the urban scenario, where the initial Macro-averaged F1 values are clearly over 60% showing potential improvement after frequency range selection. The viability of the ANED Lo-Cap for the suburban scenario is not so clear due to the poor accuracy results (slightly higher than 50%); deep frequency selection experiments should be conducted to observe whether it is worth implementing it for the suburban scenario or not with the considered classification principle adaptation.

### 6.2. ANED Lo-Cap and Network Homogeneity

The original idea of the ANED Lo-Cap design was based on the deconstruction of the phases of the ANED Hi-Cap algorithm [15], as the feature extraction, or the classification algorithm, in order to discard those that would involve a high computational cost, as well as maximizing the similarity of the classification approach considered by the original Hi-Cap version.

The study of the spectral differences between the ANE and the RTN [22] allows us to conclude that, for each acoustic scenario, the separability of the two types of signal is possible, especially if the potential algorithm focuses on specific frequency ranges, as observed in Section 4.2. The two evaluated acoustic scenarios, urban and suburban, do not present the same probability of error results in terms of separation of ANE and RTN and its associated MFS.

The analysis of the probability of error in the suburban scenario shows that the most distinctive spectral range correspond to the mid-frequencies (from 2 kHz to 2700 Hz approximately), while the lower discriminating ones comprise higher frequencies (from 3500 Hz to 24 kHz). In the suburban scenario, we mainly found three types of ANE related to natural phenomena (rain, wind and thunder), and four types of ANE [46]: sirens, horns, brakes and birds. All the most typical ANEs in the suburban scenario present spectral activity in the mid-frequencies and some of them even in the high-frequencies; therefore, its reasonable that the most accurate frequency range to distinguish between ANE from RTN in this scenario corresponds to that group of frequencies.

The results of the probability of error in the urban scenario show clear differences in terms of the spectral energy distribution in comparison with the suburban scenario. The most discriminating bands in this scenario correspond to the low-frequency region (from 33 Hz to 1 kHz), which is the range of frequencies where most of the RTN energy is located. The types of ANE found in an urban environment, apart from the natural phenomena which are the same as in the suburban scenario, are wider than the ones found in the suburban environment [46]: airplanes, doors, people talking, tramways, trains, etc. Some of these ANEs have wider distribution of spectral energy, including low, mid and high frequencies. Airplanes, for example, have their spectral energy distribution centred in the low-frequencies, and so do trams and trains (together with the mid-frequencies). Thus, the low frequency range could be potentially the best discriminating spectral region for the urban environment, which should be confirmed with further experiments.

Nevertheless, the analyses conducted over the MFS encourages us to analyze fully the probability of error depending on the selected frequency region, in order to improve the accuracy results presented in Section 6.1 as well as to maximize the efficiency of the algorithm using the minimum required spectral integration.

### 6.3. Real-Time Implementation in a Low-Cost Platform

In Section 5, it has been concluded that a commercial platform is able to support the computational load of the ANED Lo-Cap. However, not all boards are capable of processing the audio and making a decision in real time, as the cheapest option μC-based Arduino. Although this development board may be able to sample the audio, compute the FFT and apply the subband filtering, it would not be done in real-time. This condition requires that the FFT and the filtering process are conducted at the same time a new frame is read, implying that the μC should be programmed in a cooperative mode jumping sequentially between the ADC read and the FFT computation. It is possible to add an ad-hoc hardware that reads the audio and puts it in a buffer, easing the task of the μC to read at the sampling frequency. However, the system would still need to compute the FFT and take a decision within the 30 ms-frame rate. As a consequence, we discard this option to implement the ANED Lo-Cap due to the basic computational features that the platform offers.

Other hardware platforms, with higher capacity but not so higher price, offer a viable possibility to run the algorithm real-time. These platforms are mainly built around an ARM processor and usually include ad-hoc peripherals, as timers, ADCs, Ethernet interface and several (even dozens) of General-Purpose Input/Output (GPIOs). One of the most used board within this group is the Raspberry Pi 3 (from $35), which offers the possibility of audio acquisition (through the audio input) and computing the FFT in real time. In the future, several tests should be conducted to study the capability of the board and to find out the maximum number of points of the FFT that it is able to compute as well as the number of subbands.

In Section 5.3, other boards with high-speed internal ADC have been also studied, concluding that they could present a good efficiency in the implementation of the ANED Lo-Cap, e.g., NXP and STM, but at higher cost. The boards in this group have a starting price of $69 and have more specifications than the Raspberry Pi, however, these platforms are also larger, more expensive and with higher power consumption. These boards could implement both the ANED Lo-Cap and Hi-Cap, as they offer more computational capacity. Finally, the FPGA-based hardware platforms have also been analyzed and could be a suitable solution, thanks to the different programming paradigm, closer to hardware real-time signal processing restrictions. However, due to the complexity of the FPGA development boards and the particular specifications they have, they are in a higher price range, with the Arty S7 starting at $109. In this case, the Hi-Cap version of the ANED could be also implemented, as the FFT is a very common operation for the FPGA-based platforms and they do not have problems of parallelization.

In this work, the computational load of the ANED Lo-Cap has been studied in order to mount it using a commercial low-cost platform. However, other factors would influence a final implementation choice, as size, power consumption and supply, and compatibility with other peripherals depending on the needs of the project. Future research will include testing the algorithm over several hardware platforms proposed in this paper and evaluating the power consumption and performance.

## 7. Conclusions

In this work, we have analyzed the viability of an algorithm to implement an ANED Lo-Cap to run real-time in the Lo-Cap acoustic nodes of an hybrid WASN, following the same principle used for implementing the original ANED version designed for the high-capacity nodes. To that effect, the proposal is based on parametrizing the input acoustic signal with its Mel-based spectral energy distribution, and classified as RTN or ANE by means of a one-band threshold-based linear discriminator. The experiments have been conducted considering 9h and 8 min of real-life acoustic data from the suburban and urban pilot areas of the DYNAMAP project.

The main conclusion of the analysis conducted in this research is that the ANED Lo-Cap proposal is viable, both in terms of computational load and classification accuracy (at least, in the urban environment), maintaining a consistent performance with respect to the obtained by the Hi-Cap counterpart (i.e., higher Macro-averaged F1 measures for the urban environment than for the suburban scenario). From the computational analyses, we can conclude that the ANED Lo-Cap – that requires around 16 of the computational load of the ANED Hi-Cap – cannot be implemented in the most basic platforms (e.g., Arduino), but the first upgrade of low-cost and Lo-Cap hardware, with small budget differences, can assume the ANED Lo-Cap running real-time (e.g., Raspberry Pi 3). Obviously, any platform with more computational capacity, such as FPGA, can assume the ANED Lo-Cap and even the ANED Hi-Cap. However, the use of high-performance computing platforms would increase the cost of the WASN, when one of the main goals is to deploy a low-cost wireless acoustic sensor network.

Regarding the classification accuracy of the ANED Lo-Cap, the research conducted in this work concludes that the first results in both scenarios decrease the F1 Macro-averaged measure in around 9–10% in comparison with the ANED Hi-Cap, and they need further optimization. Nevertheless, the performance of the ANED Lo-Cap proposal results in both suburban and urban environments is homogeneous with its own performance in ANED Hi-Cap results [15]. In the application in the suburban environment, considering the full Mel-based frequency range, they present values slightly higher than 50% in terms of Macro-averaged F1 measure. If these results cannot be improved, the usefulness of the baseline ANED Lo-Cap in this environment will be questioned. On the contrary, the application of the ANED Lo-Cap working with all the MFS as a baseline in the urban environment shows significantly better results (higher than 60% in terms of Macro-averaged F1 measure), which encourages us to keep working in order to optimize the frequency range selection to improve the obtained results. In this direction, future work will be focused on the study of optimum frequency ranges that allow obtaining improved accuracy values, which now state around 4% lower in the full-band preliminary tests. After that, the subsequent majority voting scheme will be included so as to provide the same output scheme of the ANED Hi-Cap every one second, following the DYNAMAP project requirements for real-life operation.

## Figures and Tables

**Table 1:** Relation between the M=48 subbands used from the Mel filter-bank parameterization and its central frequency in Hz.

# of Subband	Freq. (Hz)	# of Subband	Freq. (Hz)	# of Subband	Freq. (Hz)	# of Subband	Freq. (Hz)
1	86.7	13	886.7	25	2484.6	37	7231.5
2	153.3	14	953.3	26	2715.9	38	7904.8
3	220	15	1020	27	2968.8	39	8640.9
4	286.7	16	1115	28	3242.2	40	9445.4
5	353.3	17	1218.8	29	3547.4	41	10,324.9
6	420	18	1332.3	30	3877.7	42	11,286.3
7	486.7	19	1456.3	31	4238.7	43	12,337.2
8	553.3	20	1591.9	32	4633.4	44	13,485.9
9	620	21	1740.2	33	5064.9	45	14,741.6
10	686.7	22	1902.2	34	5536.5	46	16,114.3
11	753.3	23	2079.3	35	6052	47	17,614.7
12	820	24	2272.9	36	6615.5	48	19,254.8

**Table 2:** Computational cost analysis of the audio processing.

	Additions	Multiplications	Floating Point Operations
Windowing	0	*N*	*N*
FFT	$N\cdot log_{2}\left(N\right)$	$\frac{N}{2}\cdot log_{2}\left(N\right)$	$\frac{3}{2}N\cdot log_{2}\left(N\right)$
Subband filtering	C−1	*C*	2·C−1

**Table 3:** Price comparison for the hardware platforms described and algorithm they can assume real-time.

Hardware Platform	Base Price	Supported ANED Version
Arduino Uno R3	from $20	None
Raspberry Pi Model A+	from $20	ANED Lo-Cap
NXP Semiconductor FRDM-K66F Freedom Board	from $69	ANED Lo-Cap & Hi-Cap
Arty S7: Spartan-7 FPGA	from $109	ANED Lo-Cap & Hi-Cap

## Abbreviations

The following abbreviations are used in this manuscript:

ADC	Analog to Digital Converter
AED	Acoustic Event Detection
ARM	Advanced RISC Machine
ANE	Anomalous Noise Event
ANED	Anomalous Noise Event Detection
CNOSSOS	Common Noise Assessment Methods
CPU	Central Processing Unit
DCT	Discrete Cosine Transform
DFT	Discrete Fourier Transform
DYNAMAP	DYNamic Acoustic MAPping
EC	European Commission
END	Environmental Noise Directive
EU	European Union
FFT	Fast Fourier Transform
FPGA	Field-Programmable Gate Array
GPU	Graphics Processing Unit
HMM	Hidden Markov Models
IIR	Infinite Impulse Response
LDD	Low Level Descriptors
MFCC	Mel-Frequency Cepstral Coefficients
MFS	Mel Frequency Subband
MSPS	Mega Samples per Second
OCC	One-Class Classifier
PWP	Perceptual Wavelet Packets
RTN	Road Traffic Noise
SNR	Signal to Noise Ratio
SVM	Support Vector Machine
UGMM	Universal GMM
WASN	Wireless Acoustic Sensor Network
ZCR	Zero Crossing Rate
